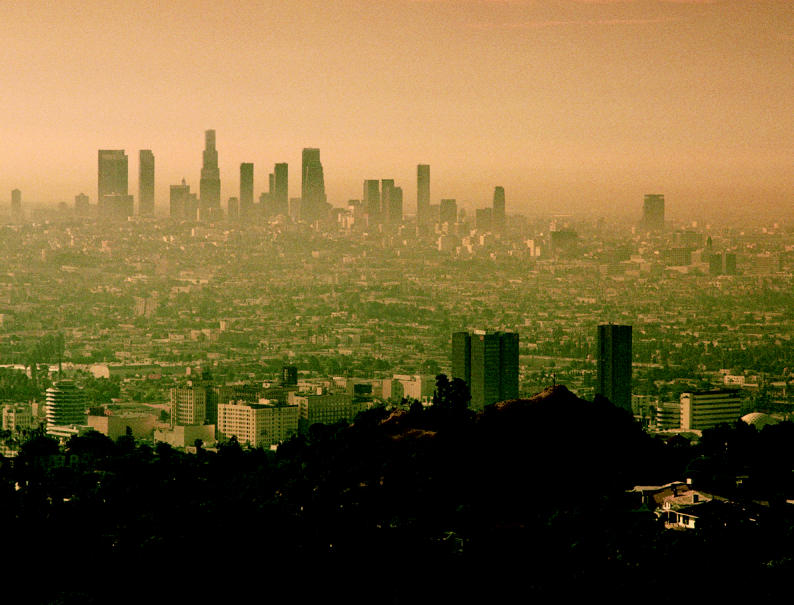# Headliners: Respiratory Health: Air Pollution Impairs Lung Development in Children

**Published:** 2004-12

**Authors:** Jerry Phelps

Gauderman WJ, Avol E, Gilliland F, Vora H, Thomas D, Berhane K, McConnell R, Kuenzli N, Lurmann F, Rappaport E, Margolis H, Bates D, Peters J. 2004. The effect of air pollution on lung development from 10 to 18 years of age. N Engl J Med 351(11):1057–1067.

Mounting evidence suggests that exposure to air pollution has long-term effects on lung development in children; reductions in lung function have been observed in studies in Europe and the United States. To further investigate these effects, this NIEHS-supported research team performed a prospective epidemiologic study on 1,759 children from 12 communities in Southern California.

The communities had a wide range of exposures to air pollutants including particulate matter, acid aerosols, ozone, and nitrogen dioxide. The team recruited fourth-graders and performed lung function tests annually for eight years.

Over the eight-year period, decreases in a measurement of lung function known as forced expiratory volume (FEV_1_) were associated with exposure to nitrogen dioxide, acid aerosols, particulate matter, and elemental carbon. The decreases noted were statistically and clinically significant. For example, the risk of diminished FEV_1_ was almost five times higher at the highest level of particulate matter exposure than at the lowest level. The magnitude of the effects on development of lung function was comparable to that reported for exposure to maternal smoking.

The authors conclude that these results can be generalized to children living in other parts of the United States that have high air pollution levels. The results indicate that current ambient air pollution levels can have chronic and adverse effects on lung development in children, leading to clinically significant lung function deficits in adulthood. Given the severity of the effects and the importance of lung development as a determinant of morbidity and mortality during adulthood, it is important to continue identifying strategies for reducing air pollution.

## Figures and Tables

**Figure:**